# 

**Published:** 1864-01

**Authors:** 


### INDEX

TO

### HALL'S JOURNAL OF HEALTH.

### VOL. XI, 1864.

| | PAGE |
|---|---|
| Apoplexy, | 67 |
| Admonishing, | 76 |
| Agricultural colleges, | 90 |
| Asthma, | 139 |
| Biliousness, | 231, 20 |
| Bread, | 37, 45 |
| Bronchitis, | 125 |
| Business success, | 146 |
| " Retiring from, | 156 |
| Consumption, | 25 |
| " Climate for, | 31 |
| " its beginnings, | 192 |
| Corn Bread, | 37 |
| Children corrected, | 39 |
| " Ingratitude of, | 78 |
| " of the Rich, | 171 |
| " Dirty, | 202 |
| Convenient knowledge, | 40 |
| Charms, | 42 |
| Cell development, | 137 |
| Croup, | 139 |
| Christianity Joyous, | 160 |
| Checking Perspiration, | 163 |
| Colds how taken, | 163 |
| Cooking Meats, | 164 |
| " Economical, | 165 |
| Cough Hacking, | 192 |
| Cattle Feeding, | 198 |
| Central Park, | 207 |
| Cholera, | 229 |
| Deranged, | 38 |
| Death, | 58 |
| Drowning, | 82 |
| Dress, | 77, 118 |
| Drinks, Invalid | 140 |
| Dodge's Tincture, | 226 |
| Diarrhoea, | 228 |
| Dysentery, | 230 |
| Entering a Room, | 79 |
| Eating, | 19, 43, 87, 179, 180 |
| Economy a Duty, | 103 |
| Flies Destroyed, | 18 |
| Frenzy, | 56 |
| Fidelity, | 60 |
| Flowers in Winter, | 80 |
| Families Saved, | 111 |
| Food and Health, | 114 |
| " Value of, | 181 |
| " Digestibility, | 185 |
| " Nutritiousness, | 186 |
| " Elements, | 200 |
| Feet, Fetid, | 138 |
| Fortunes Made, | 197 |
| Fuel Saved, | 165 |
| " Selection of, | 201 |
| Glue Liquid, | 20 |
| Growing Old, | 79 |
| Great Men's Sufferings, | 233 |
| Health and Music, | 1 |
| Head Ache, | 19 |
| How to rise, | 63 |
| Household Knowledge, | 89 |
| Happiest Persons, | 109 |
| Hunger, | 184 |
| Hacking Cough, | 192 |
| Horse Rations, | 199 |
| Health and Gold, | 237 |
| Husband, the Kind, | 240 |
| Home, a Pleasant, | 248 |

PAGE
In the Mind, .................... 41
Inheritances, ....................115
Imprisonment, ....................134
Ice, its uses, ....................165
Investments, Permanent, ..........239

Kindness Rewarded, .............. 36

Liquid Glue, .................... 20
Life Uncertain, ................. 81
Liquid Measures, .................140
Living Beyond Means, .............175
Lice in Cattle and Hair, .........226
Loose Bowels, ....................228

Music and Health, ............... 1
Mind and Body, .................. 44
" In the, ....................... 41
" Rest of, ...................... 83
Marriage, ...................249, 71
Mother, ......................61, 73
Melodies, ........................168
Mitchell the Astronomer, .........241
Milton's Habits, .................246

Never, .......................... 69
Nepevthe, ........................113

Over Eating, .................... 19

Open Fire Places, ...........236, 141

Old Man's Story, ................ 50

Paste, .......................... 20
Parental Corrections, ........... 47
Philosophy, ..................... 75
Prejudice, ...................... 76
Piano Forte, .....................143
Potatoes, ........................181
Perseverance, Indomitable, .......241

Recuperation, ................... 32
Resurrection Flower, ............ 80
Restless Nights, .................116

Sick Head Ache, ................. 19
Skating, ........................ 24
School Girls, ................... 35

PAGE
Stomach, ........................ 88
Sunshine, ....................... 98
Safety of Families, ..............111
Sleeplessness. ..............195, 116
Starvation, ......................133
Summer Recreations, ..............149
" Excursions, ....................162
Strange People, ..................153
Salt Rheum, ......................203
Soldier Health, ..................211
Sabbath Observance, ..............225
Soldiers, All, ...................227
School of Misses Bucknall, .......226

Teachers, Harsh, ................ 49
Tables, Valuable, ............... 72
Time, its Value, ................ 81
Tonsil Cutting, ..................137
Travelling Hints, ................166
Tomatoes, ........................192
Thirsting to Death, ..............194

White Wash, ..................... 19
Weather and Wealth, ............. 70
Wedding, First, ................. 71
War, Philosophy of .............. 93
Wakefulness, ................116, 195
Worth Knowing, ...................201
Warmth and Strength, .............202
Washington. ......................247

POETRY.

Poets and Music, ................ 1
Anvil Strike, ................... 14
Birds in May, ................... 9
Boy that Died, ...................117
Darkness and Light, ............. 17
Going Alone, .................... 16
Heaven, ......................... 11
Milton's Blindness, ............. 15
Neighbor, ....................... 14
Over the River, ................. 10
Skeleton, ....................... 12
School Boy Days, ................ 13
The Rest, ........................117

### INDEX TO 182 HEALTH TRACTS.

NO. OF TRACT

Air and Sunshine,................ 51
Apples,.......................... 59
Antidotes of Poisons,............134

Baldness,........................124
Bathing,......................... 30
Beards,.......................... 74
Bites,.........................23, 27
Bilious Diarrhea,................153
Burns,.................23, 27, 80, 129

Colds, Cured,...................3, 27
" Neglected,............27, 29, 36
" Prevented, ................ 31
" Catching,..................144
Checking Perspiration ............ 58
Catarrh,......................... 52
Corns and Shoes,................. 14
Coffee,..................46, 72, 128
Costiveness,..................... 22
'Cute Things,....................120
Children at School,..............132
Cures,...........................135
Changing Clothing,...............141
Cholera,.........................151
Clergymen, Saving,...............158
Cancer,..........................177

Drunkenness,..................... 8
Dyspepsia,.....................8, 33
Disease, Its Causes,............. 4
Disease Avoided,................. 28
Disease not Causeless,...........164
Drinking,........................ 35
Diet for Invalids,............... 54
Deafness,........................ 70
Dying Easily,....................143
Drowning,........................146
Dieting,.........................147
Diphtheria,......................149
Diarrhea,........................150
Dysentery,.......................152
Disinfectants,...................154
Death Rate,......................159

Erect Position, ................. 11
Eating,.......................26, 171
Eating Wisely,................... 32

NO. OF TRACT

Eat, How to...................... 34
Eating Habits,...................142
Eyes, Care of, .................5, 15
Eyesight Failing,................ 61
Erysipelas, ..................... 56
Escaping from Fire,..............157
Emanations,......................145
Exercise, .......................174

Fruits in Summer,................ 2
Flannel Wearing,..............19, 130
Feet of Children, ..........14, 21, 180
Fifteen Follies, ................ 53
Fire Escape,.....................157
Fifth Avenue Sights,.............153

Growing Beautiful,............... 15
Great Eaters,....................171
Genius, its Vices, ..............161

Hair,............................ 18
Headache, ....................41, 62
Health without Medicine,......... 20
Healthful Observances,........... 38
Health Essentials,............... 42
Hydrophobia, .................... 49
Housekeeping, ...........75, 94, 122, 144
Health Theories,................. 77

Inconsiderations,................ 1
Ice, its Uses,................... 9
Inverted Toe-Nail,............... 64
Insanity,........................173

Life Wasted,.....................138
Loose Bowels,....................150
Leaving Home,....................162
Logic Run Mad, ..................172

Music Healthful,...............7, 81
Medicine, Taking,................ 60
Memories, ....................... 71
Milk, its Uses, ................. 82
Medical Items,................169, 182
Month Malign,....................170

Nothing but a Cold, ............. 36

Neuralgia, ...................... 44

| | NO. OF TRACT |
|---|---|
| One Acre, | 139 |
| One by One, | 160 |
| Obscure Diseases, | 165 |
| Precautions, | 37 |
| Presence of Mind, | 39 |
| Premonitions, | 43 |
| Private Things, | 45 |
| Poisons, | 23, 27, 55, 134 |
| Physiological Aphorisms, | 65 |
| Pain, | 67 |
| Potatoes, | 123 |
| Philosophy, | 137 |
| Physiological Items, | 155 |
| Posture in Worship, | 176 |
| Rheumatism, | 50 |
| Read and Heed, | 69 |
| Resignation, | 145 |
| Sitting Erect, | 13 |
| Sabbath, | 17, 156 |
| Scalds, | 80 |
| School-Children, | 132, 183 |
| Sour Stomach, | 24 |
| Sleeping, | 25 |
| Skating, | 63 |
| Suppers, Light, | 73 |
| Summering, | 78 |

| | NO. OF TRACT |
|---|---|
| Spot, The One, | 126 |
| Specifics, | 138 |
| Spring-Time, | 140 |
| Summer Drinks, | 148 |
| Saving Ministers, | 158 |
| Stammering, | 179 |
| Soups and Gruels, | 181 |
| Traveling Hints, | 6 |
| The Three P's, | 47 |
| Teeth, | 68 |
| Urination, | 66 |
| Valuable Knowledge, | 27 |
| Vaccination, | 79 |
| Ventilation, | 125 |
| Vermin, Household, | 136 |
| Vices of Genius, | 161 |
| Winter Rules, | 11 |
| Walking, | 18 |
| Warnings, | 48 |
| Worship, Religious, | 168 |
| Woolen Clothing, | 19, 134 |
| White Washes, | 134 |
| Worth Remembering, | 167 |
| Weather Signs, | 172 |

All the above Health Tracts, with a great variety of other practical reading in reference to Health, will be found in the two volumes for 1862 and 1863, $1.25 each, both for $2, at the office, 831 Broadway, New-York.

